# 2-Amino-4-(4-meth­oxy­phen­yl)-7,7-di­methyl-5-oxo-5,6,7,8-tetra­hydro-4*H*-chromene-3-carbonitrile propan-2-one monosolvate

**DOI:** 10.1107/S160053681202781X

**Published:** 2012-06-27

**Authors:** Shaaban K. Mohamed, Mehmet Akkurt, Muhammad N. Tahir, Antar A. Abdelhamid, M. A. Allahverdiyev

**Affiliations:** a Chemistry and Environmental Division, Manchester Metropolitan University, Manchester M1 5GD, England; b Department of Physics, Faculty of Sciences, Erciyes University, 38039 Kayseri, Turkey; c University of Sargodha, Department of Physics, Sargodha, Pakistan; d Department of Organic Chemistry, Baku State University, Baku, Azerbaijan

## Abstract

In the crystal structure of the title compound, C_19_H_20_N_2_O_3_·C_3_H_6_O, mol­ecules are linked into inversion dimers with an *R*_2_^2^(12) motif by pairs of N—H⋯N hydrogen bonds. These dimers are further connected into chains running along the *a* axis by N—H⋯O hydrogen bonds. C—H⋯N and C—H⋯π inter­actions also feature in the packing. The cyclohexene ring adopts nearly an envelope conformation [puckering parameters are *Q*_T_ = 0.456 (2) Å, θ = 54.6 (3)° and ϕ = 225.2 (3)°].

## Related literature

For pharmacological properties of 4*H*-chromene and fused 4*H*-chromene derivatives, see: Kemnitzer *et al.* (2007 ▶, 2008 ▶); Abd-El-Aziz *et al.* (2004 ▶, 2007 ▶); Sabry *et al.* (2011 ▶); Gourdeau *et al.* (2004 ▶); Mahdavi *et al.* (2011 ▶); Brühlmann *et al.* (2001 ▶). For a related structure, see: Mohamed *et al.* (2012 ▶). For puckering parameters, see: Cremer & Pople (1975 ▶). For hydrogen-bond motifs, see: Bernstein *et al.* (1995 ▶).
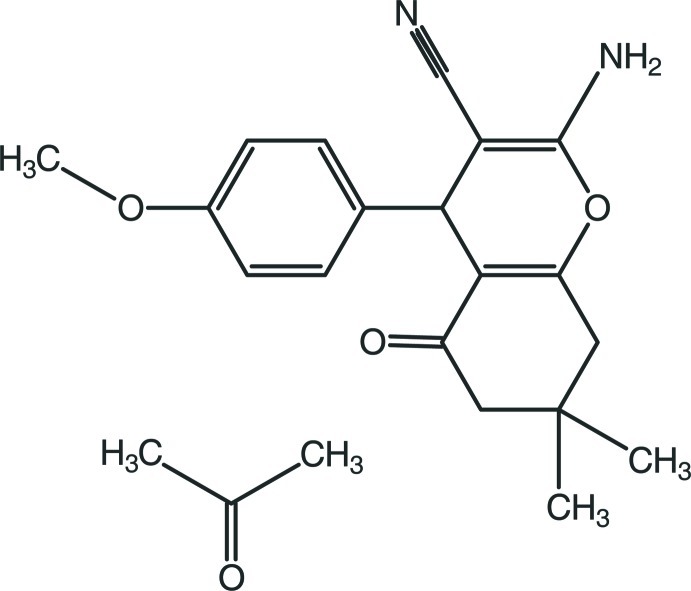


## Experimental

### 

#### Crystal data

C_19_H_20_N_2_O_3_·C_3_H_6_O
*M* *_r_* = 382.45
Triclinic, 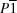

*a* = 8.2037 (4) Å
*b* = 9.5319 (4) Å
*c* = 13.8390 (7) Å
α = 77.743 (2)°
β = 87.307 (3)°
γ = 80.672 (2)°
*V* = 1043.43 (9) Å^3^
*Z* = 2
Mo *K*α radiation
μ = 0.08 mm^−1^
*T* = 296 K
0.28 × 0.22 × 0.20 mm

#### Data collection

Bruker Kappa APEXII CCD diffractometer
Absorption correction: multi-scan (*SADABS*; Bruker, 2005 ▶) *T*_min_ = 0.978, *T*_max_ = 0.983
16752 measured reflections
4917 independent reflections
3284 reflections with *I* > 2σ(*I*)
*R*_int_ = 0.027

#### Refinement

*R*[*F* ^2^ > 2σ(*F* ^2^)] = 0.059
*wR*(*F* ^2^) = 0.182
*S* = 1.04
4917 reflections
246 parameters
1 restraint
H-atom parameters constrained
Δρ_max_ = 0.59 e Å^−3^
Δρ_min_ = −0.50 e Å^−3^

### 

Data collection: *APEX2* (Bruker, 2007 ▶); cell refinement: *SAINT* (Bruker, 2007 ▶); data reduction: *SAINT*; program(s) used to solve structure: *SHELXS97* (Sheldrick, 2008 ▶); program(s) used to refine structure: *SHELXL97* (Sheldrick, 2008 ▶); molecular graphics: *ORTEP-3 for Windows* (Farrugia, 1997 ▶) and *PLATON* (Spek, 2009 ▶); software used to prepare material for publication: *WinGX* (Farrugia, 1999 ▶) and *PLATON*.

## Supplementary Material

Crystal structure: contains datablock(s) global, I. DOI: 10.1107/S160053681202781X/bt5943sup1.cif

Structure factors: contains datablock(s) I. DOI: 10.1107/S160053681202781X/bt5943Isup2.hkl

Supplementary material file. DOI: 10.1107/S160053681202781X/bt5943Isup3.cml

Additional supplementary materials:  crystallographic information; 3D view; checkCIF report

## Figures and Tables

**Table 1:** Hydrogen-bond geometry (Å, °) *Cg*2 is the centroid of the O2/C8–C10/C12/C13) 4*H*-pyran ring.

*D*—H⋯*A*	*D*—H	H⋯*A*	*D*⋯*A*	*D*—H⋯*A*
N2—H2*A*⋯N1^i^	0.86	2.30	3.142 (3)	168
N2—H2*B*⋯O3^ii^	0.86	2.19	3.029 (2)	165
C6—H6⋯N1^iii^	0.93	2.51	3.258 (3)	137
C18—H18*A*⋯*Cg*2^iv^	0.96	2.77	3.677 (2)	158

Symmetry codes: (i) 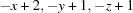; (ii) 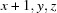; (iii) 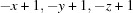; (iv) 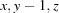.

**Fig. 1.**
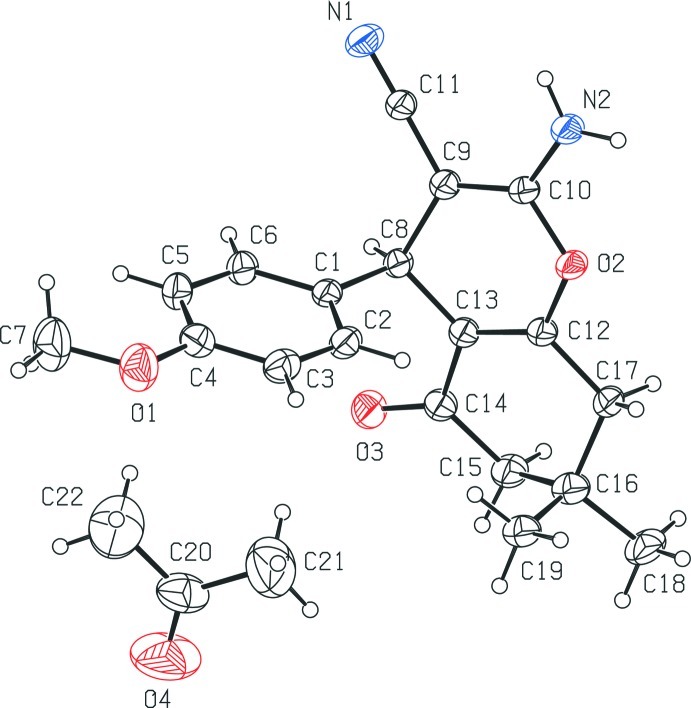
The molecular structure of the title compound with the atom numbering scheme. Displacement ellipsoids for non-H atoms are drawn at the 30% probability level.

**Fig. 2.**
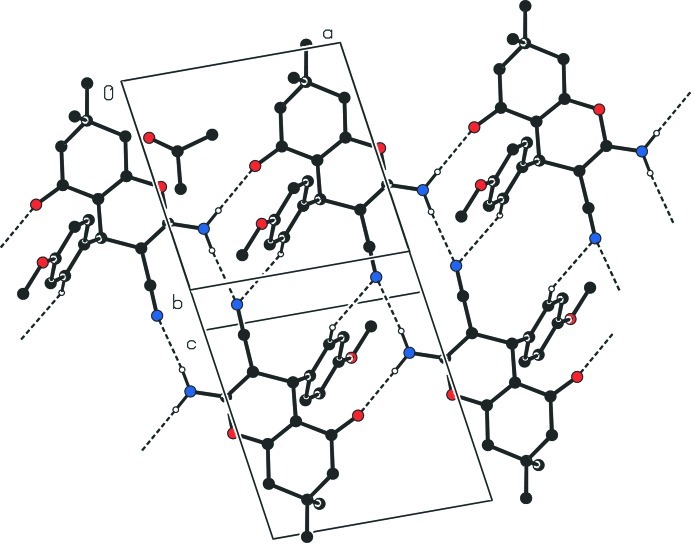
View of the dimers formed by pairs of N—H···N hydrogen bonds, with an R22(12) motif and the N—H···O and C—H···N hydrogen bonds connect the dimers with each other. H atoms not involved in hydrogen bonds have been omitted for clarity.

## supplementary crystallographic information

### Comment

Compounds such as 4*H*-chromenes and fused 4*H*-chromenes are a great class of organic drugs due to their wide applications in chemo therapy. They exhibited anticancer activities (Kemnitzer *et al.*, 2007, 2008; Abd-El-Aziz *et al.*, 2004, 2007; Gourdeau *et al.*, 2004; Sabry *et al.*, 2011; Mahdavi *et al.*, 2011) and have used in treatment of Alzheimer's disease and Schizophrenia disorder (Brühlmann *et al.*, 2001). In this context and further to our on-going study in synthesis and biological investigation of 4*H*-chromene compounds (Mohamed *et al.* 2012), we report in this study the structure and synthesis of the title compound (I).

In the title compound (I) shown in Fig. 1, the C12–C17 cyclohexene and O2/C8—C10/C12/C13 4*H*-pyran rings are puckered with the puckering parameters (Cremer & Pople, 1975) of Q_T_ = 0.456 (2) ° A, θ = 54.6 (3) °, φ = 225.2 (3) °, and Q_T_ = 0.201 (2) Å, θ = 108.1 (5) °, φ = 354.7 (6) °, respectively. The bond lengths and bond angles in (I) are normal and are comparable with those of a related structure previously reported (Mohamed *et al.*, 2012).

In the crystal structure, adjacent molecules are connected by the pairs of N—H···N hydrogen bonds, forming dimers, with an *R*^2^_2_(12) motif (Bernstein *et al.*, 1995; Table 1, Fig. 2). These dimers are further connected to chains running along the a axis by N—H···O hydrogen bonds. Furthermore, C—H···π interactions contribute to the stabilization of the crystal packing (Table 1, Fig. 2).

### Experimental

To a solution of 184 mg (1 mmol) (4-methoxybenzylidene)propanedinitrile in 50 ml ethanol, 140 mg (1 mmol) 5,5-dimethylcyclohexane-1,3-dione was added in presence of 123 mg (1 mmol) (4-aminophenyl)methanol as catalyst. The reaction mixture was refluxed at 350 K for 5 h, then cooled at ambient temperature. The resin product that formed was solidified by washing with acetone then filtered off under vacuum and recrystallized from ethanol. Single crystals suitable for X-ray diffraction were produced by slow evaporation of ethanol/acetone solution (1:1) of (I) over two days at room temperature. Yield is 86%, *M*.p. 443 K.

### Refinement

All H atoms were positioned geometrically and refined by using a riding model, with N—H = 0.86 Å and C—H = 0.93 Å (aromatic), 0.96 Å (methyl), 0.97 Å (methylene) and 0.98 Å (methine), with *U*_iso_(H) = 1.5*U*_eq_(O) for methyl groups and *U*_iso_(H) = 1.2*U*_eq_(C, N) for others.

### Crystal data

**Table:**

C_19_H_20_N_2_O_3_·C_3_H_6_O	*Z* = 2
*M**_r_* = 382.45	*F*(000) = 408
Triclinic, *P*1	*D*_x_ = 1.217 Mg m^−^^3^
Hall symbol: -P 1	Mo *K*α radiation, λ = 0.71073 Å
*a* = 8.2037 (4) Å	Cell parameters from 360 reflections
*b* = 9.5319 (4) Å	θ = 21.3–3.4°
*c* = 13.8390 (7) Å	µ = 0.08 mm^−^^1^
α = 77.743 (2)°	*T* = 296 K
β = 87.307 (3)°	Prism, white
γ = 80.672 (2)°	0.28 × 0.22 × 0.20 mm
*V* = 1043.43 (9) Å^3^	

### Data collection

**Table:**

Bruker Kappa APEXII CCD diffractometer	4917 independent reflections
Radiation source: fine-focus sealed tube	3284 reflections with *I* > 2σ(*I*)
Graphite monochromator	*R*_int_ = 0.027
Detector resolution: 0.81 pixels mm^-1^	θ_max_ = 28.0°, θ_min_ = 2.2°
ω scans	*h* = −10→10
Absorption correction: multi-scan (*SADABS*; Bruker, 2005)	*k* = −12→9
*T*_min_ = 0.978, *T*_max_ = 0.983	*l* = −18→18
16752 measured reflections	

### Refinement

**Table:**

Refinement on *F*^2^	Primary atom site location: structure-invariant direct methods
Least-squares matrix: full	Secondary atom site location: difference Fourier map
*R*[*F*^2^ > 2σ(*F*^2^)] = 0.059	Hydrogen site location: inferred from neighbouring sites
*wR*(*F*^2^) = 0.182	H-atom parameters constrained
*S* = 1.04	*w* = 1/[σ^2^(*F*_o_^2^) + (0.0878*P*)^2^ + 0.2431*P*] where *P* = (*F*_o_^2^ + 2*F*_c_^2^)/3
4917 reflections	(Δ/σ)_max_ < 0.001
246 parameters	Δρ_max_ = 0.59 e Å^−^^3^
1 restraint	Δρ_min_ = −0.50 e Å^−^^3^

### Special details

**Table:**

Geometry. Bond distances, angles *etc*. have been calculated using the rounded fractional coordinates. All su's are estimated from the variances of the (full) variance-covariance matrix. The cell e.s.d.'s are taken into account in the estimation of distances, angles and torsion angles
Refinement. Refinement on *F*^2^ for ALL reflections except those flagged by the user for potential systematic errors. Weighted *R*-factors *wR* and all goodnesses of fit *S* are based on *F*^2^, conventional *R*-factors *R* are based on *F*, with *F* set to zero for negative *F*^2^. The observed criterion of *F*^2^ > σ(*F*^2^) is used only for calculating -*R*-factor-obs *etc*. and is not relevant to the choice of reflections for refinement. *R*-factors based on *F*^2^ are statistically about twice as large as those based on *F*, and *R*-factors based on ALL data will be even larger.

### Fractional atomic coordinates and isotropic or equivalent isotropic displacement parameters (Å^2^)

**Table:**

	*x*	*y*	*z*	*U*_iso_*/*U*_eq_	
O1	0.4032 (2)	0.66247 (19)	0.08006 (13)	0.0802 (7)	
O2	1.02042 (14)	0.08753 (13)	0.35411 (10)	0.0488 (4)	
O3	0.45650 (17)	0.03885 (16)	0.37857 (13)	0.0629 (6)	
N1	0.8196 (2)	0.4700 (2)	0.51973 (16)	0.0656 (7)	
N2	1.14500 (19)	0.24295 (18)	0.41136 (14)	0.0563 (6)	
C1	0.6070 (2)	0.33700 (18)	0.31182 (14)	0.0399 (5)	
C2	0.6619 (2)	0.3463 (2)	0.21430 (15)	0.0483 (6)	
C3	0.5919 (3)	0.4548 (2)	0.13983 (16)	0.0543 (7)	
C4	0.4642 (3)	0.5588 (2)	0.16012 (16)	0.0532 (7)	
C5	0.4076 (2)	0.5523 (2)	0.25594 (17)	0.0549 (7)	
C6	0.4797 (2)	0.4416 (2)	0.33068 (15)	0.0473 (6)	
C7	0.2741 (4)	0.7714 (3)	0.0945 (2)	0.0952 (11)	
C8	0.6896 (2)	0.22102 (18)	0.39559 (14)	0.0389 (5)	
C9	0.8474 (2)	0.26366 (19)	0.42629 (14)	0.0437 (4)	
C10	0.9993 (2)	0.20384 (19)	0.39964 (14)	0.0419 (5)	
C11	0.8333 (2)	0.37739 (19)	0.47771 (14)	0.0437 (4)	
C12	0.8867 (2)	0.02105 (18)	0.34609 (14)	0.0408 (5)	
C13	0.7325 (2)	0.07535 (18)	0.36796 (13)	0.0403 (5)	
C14	0.5994 (2)	−0.00856 (19)	0.36226 (15)	0.0455 (6)	
C15	0.6483 (2)	−0.1573 (2)	0.33914 (17)	0.0538 (7)	
C16	0.7957 (3)	−0.1685 (2)	0.26744 (17)	0.0570 (5)	
C17	0.9388 (2)	−0.11592 (19)	0.30923 (16)	0.0475 (6)	
C18	0.8500 (3)	−0.3269 (2)	0.2591 (2)	0.0702 (9)	
C19	0.7493 (3)	−0.0761 (2)	0.16615 (17)	0.0570 (5)	
O4	0.0402 (4)	0.1650 (4)	0.0951 (3)	0.1652 (16)	
C20	0.1432 (4)	0.2327 (4)	0.1086 (2)	0.0875 (11)	
C21	0.3163 (5)	0.1707 (5)	0.1206 (4)	0.149 (2)	
C22	0.1030 (5)	0.3830 (4)	0.1273 (4)	0.142 (2)	
H2	0.74790	0.27740	0.19950	0.0580*	
H2A	1.14960	0.31490	0.43900	0.0680*	
H2B	1.23420	0.19620	0.39120	0.0680*	
H3	0.63040	0.45860	0.07520	0.0650*	
H5	0.32180	0.62150	0.27050	0.0660*	
H6	0.44110	0.43780	0.39530	0.0570*	
H7A	0.18230	0.72780	0.12580	0.1420*	
H7B	0.24060	0.83210	0.03180	0.1420*	
H7C	0.31060	0.82910	0.13580	0.1420*	
H8	0.61350	0.21200	0.45250	0.0470*	
H15A	0.55440	−0.18350	0.31090	0.0650*	
H15B	0.67530	−0.22710	0.40040	0.0650*	
H17A	0.98780	−0.19130	0.36310	0.0570*	
H17B	1.02270	−0.10000	0.25810	0.0570*	
H18A	0.75920	−0.36330	0.23680	0.1050*	
H18B	0.88460	−0.38430	0.32260	0.1050*	
H18C	0.94030	−0.33260	0.21270	0.1050*	
H19A	0.71430	0.02300	0.17170	0.0850*	
H19B	0.66090	−0.11140	0.14030	0.0850*	
H19C	0.84340	−0.08150	0.12240	0.0850*	
H21A	0.35150	0.17590	0.18470	0.2240*	
H21B	0.38070	0.22410	0.07060	0.2240*	
H21C	0.33130	0.07090	0.11440	0.2240*	
H22A	0.15880	0.44750	0.07910	0.2140*	
H22B	0.13850	0.38360	0.19230	0.2140*	
H22C	−0.01410	0.41450	0.12260	0.2140*	

### Atomic displacement parameters (Å^2^)

**Table:**

	*U*^11^	*U*^22^	*U*^33^	*U*^12^	*U*^13^	*U*^23^
O1	0.0853 (12)	0.0744 (11)	0.0695 (11)	0.0109 (9)	−0.0079 (9)	−0.0055 (9)
O2	0.0325 (6)	0.0517 (7)	0.0730 (9)	−0.0094 (5)	0.0028 (6)	−0.0355 (7)
O3	0.0363 (7)	0.0646 (9)	0.0955 (12)	−0.0134 (6)	0.0069 (7)	−0.0313 (8)
N1	0.0476 (10)	0.0739 (12)	0.0915 (14)	−0.0128 (9)	0.0111 (9)	−0.0534 (11)
N2	0.0338 (8)	0.0609 (10)	0.0875 (13)	−0.0101 (7)	−0.0002 (8)	−0.0433 (9)
C1	0.0310 (8)	0.0410 (9)	0.0535 (11)	−0.0093 (7)	0.0008 (7)	−0.0203 (8)
C2	0.0443 (10)	0.0474 (10)	0.0576 (12)	−0.0066 (8)	0.0043 (9)	−0.0224 (9)
C3	0.0581 (12)	0.0571 (11)	0.0512 (12)	−0.0099 (10)	0.0041 (10)	−0.0193 (9)
C4	0.0499 (11)	0.0530 (11)	0.0580 (13)	−0.0082 (9)	−0.0084 (10)	−0.0128 (9)
C5	0.0408 (10)	0.0538 (11)	0.0708 (14)	0.0020 (8)	−0.0011 (9)	−0.0216 (10)
C6	0.0367 (9)	0.0521 (10)	0.0557 (12)	−0.0056 (8)	0.0043 (8)	−0.0189 (9)
C7	0.093 (2)	0.0738 (17)	0.101 (2)	0.0192 (15)	−0.0154 (17)	−0.0002 (15)
C8	0.0314 (8)	0.0416 (9)	0.0480 (10)	−0.0077 (7)	0.0039 (7)	−0.0184 (7)
C9	0.0351 (7)	0.0473 (7)	0.0538 (8)	−0.0064 (5)	0.0003 (6)	−0.0222 (6)
C10	0.0381 (9)	0.0428 (9)	0.0505 (10)	−0.0070 (7)	−0.0030 (8)	−0.0214 (8)
C11	0.0351 (7)	0.0473 (7)	0.0538 (8)	−0.0064 (5)	0.0003 (6)	−0.0222 (6)
C12	0.0355 (9)	0.0407 (9)	0.0504 (10)	−0.0082 (7)	−0.0015 (8)	−0.0172 (8)
C13	0.0350 (9)	0.0401 (9)	0.0484 (10)	−0.0073 (7)	−0.0005 (7)	−0.0142 (7)
C14	0.0388 (10)	0.0458 (9)	0.0553 (11)	−0.0118 (8)	0.0007 (8)	−0.0145 (8)
C15	0.0479 (11)	0.0469 (10)	0.0738 (14)	−0.0187 (8)	0.0022 (10)	−0.0208 (9)
C16	0.0577 (9)	0.0558 (8)	0.0649 (9)	−0.0136 (7)	−0.0035 (7)	−0.0248 (7)
C17	0.0408 (10)	0.0410 (9)	0.0656 (13)	−0.0045 (7)	−0.0014 (9)	−0.0233 (9)
C18	0.0664 (14)	0.0530 (12)	0.1049 (19)	−0.0192 (10)	0.0059 (13)	−0.0410 (13)
C19	0.0577 (9)	0.0558 (8)	0.0649 (9)	−0.0136 (7)	−0.0035 (7)	−0.0248 (7)
O4	0.118 (2)	0.219 (3)	0.206 (3)	−0.089 (2)	0.007 (2)	−0.104 (3)
C20	0.0769 (19)	0.110 (2)	0.090 (2)	−0.0446 (17)	0.0029 (15)	−0.0312 (17)
C21	0.092 (3)	0.125 (3)	0.237 (6)	−0.014 (2)	−0.018 (3)	−0.049 (4)
C22	0.115 (3)	0.115 (3)	0.199 (5)	−0.032 (2)	0.019 (3)	−0.031 (3)

### Geometric parameters (Å, º)

**Table:**

O1—C4	1.372 (3)	C16—C18	1.531 (3)
O1—C7	1.397 (4)	C2—H2	0.9300
O2—C10	1.371 (2)	C3—H3	0.9300
O2—C12	1.373 (2)	C5—H5	0.9300
O3—C14	1.215 (2)	C6—H6	0.9300
O4—C20	1.187 (5)	C7—H7B	0.9600
N1—C11	1.144 (3)	C7—H7C	0.9600
N2—C10	1.335 (2)	C7—H7A	0.9600
N2—H2B	0.8600	C8—H8	0.9800
N2—H2A	0.8600	C15—H15B	0.9700
C1—C2	1.392 (3)	C15—H15A	0.9700
C1—C8	1.521 (3)	C17—H17A	0.9700
C1—C6	1.379 (3)	C17—H17B	0.9700
C2—C3	1.368 (3)	C18—H18B	0.9600
C3—C4	1.383 (3)	C18—H18C	0.9600
C4—C5	1.376 (3)	C18—H18A	0.9600
C5—C6	1.387 (3)	C19—H19B	0.9600
C8—C9	1.522 (2)	C19—H19C	0.9600
C8—C13	1.501 (2)	C19—H19A	0.9600
C9—C10	1.353 (2)	C20—C21	1.450 (5)
C9—C11	1.405 (3)	C20—C22	1.492 (5)
C12—C13	1.333 (2)	C21—H21A	0.9600
C12—C17	1.493 (3)	C21—H21B	0.9600
C13—C14	1.468 (2)	C21—H21C	0.9600
C14—C15	1.507 (3)	C22—H22A	0.9600
C15—C16	1.533 (3)	C22—H22B	0.9600
C16—C19	1.518 (3)	C22—H22C	0.9600
C16—C17	1.530 (3)		
			
C4—O1—C7	119.00 (19)	C1—C6—H6	119.00
C10—O2—C12	118.71 (13)	C5—C6—H6	119.00
C10—N2—H2B	120.00	O1—C7—H7A	109.00
H2A—N2—H2B	120.00	O1—C7—H7B	109.00
C10—N2—H2A	120.00	O1—C7—H7C	110.00
C6—C1—C8	120.86 (17)	H7A—C7—H7B	109.00
C2—C1—C6	117.49 (17)	H7A—C7—H7C	110.00
C2—C1—C8	121.58 (16)	H7B—C7—H7C	109.00
C1—C2—C3	121.33 (18)	C1—C8—H8	108.00
C2—C3—C4	120.4 (2)	C9—C8—H8	108.00
O1—C4—C3	115.55 (19)	C13—C8—H8	109.00
C3—C4—C5	119.50 (19)	C14—C15—H15A	109.00
O1—C4—C5	124.96 (19)	C14—C15—H15B	109.00
C4—C5—C6	119.47 (18)	C16—C15—H15A	109.00
C1—C6—C5	121.83 (18)	C16—C15—H15B	109.00
C1—C8—C13	112.40 (15)	H15A—C15—H15B	108.00
C1—C8—C9	110.68 (14)	C12—C17—H17A	109.00
C9—C8—C13	108.21 (14)	C12—C17—H17B	109.00
C8—C9—C10	122.50 (16)	C16—C17—H17A	109.00
C8—C9—C11	118.28 (15)	C16—C17—H17B	109.00
C10—C9—C11	119.04 (16)	H17A—C17—H17B	108.00
N2—C10—C9	128.41 (18)	C16—C18—H18A	109.00
O2—C10—N2	110.29 (15)	C16—C18—H18B	110.00
O2—C10—C9	121.30 (15)	C16—C18—H18C	110.00
N1—C11—C9	179.09 (19)	H18A—C18—H18B	109.00
O2—C12—C13	123.20 (16)	H18A—C18—H18C	109.00
O2—C12—C17	110.77 (14)	H18B—C18—H18C	109.00
C13—C12—C17	126.03 (16)	C16—C19—H19A	109.00
C12—C13—C14	118.86 (16)	C16—C19—H19B	109.00
C8—C13—C14	118.84 (15)	C16—C19—H19C	109.00
C8—C13—C12	122.28 (15)	H19A—C19—H19B	109.00
O3—C14—C13	121.03 (17)	H19A—C19—H19C	109.00
O3—C14—C15	121.68 (16)	H19B—C19—H19C	109.00
C13—C14—C15	117.26 (15)	O4—C20—C21	122.9 (4)
C14—C15—C16	113.98 (16)	O4—C20—C22	122.7 (4)
C17—C16—C18	108.51 (18)	C21—C20—C22	114.0 (3)
C17—C16—C19	110.37 (18)	C20—C21—H21A	109.00
C18—C16—C19	109.63 (19)	C20—C21—H21B	109.00
C15—C16—C19	110.12 (19)	C20—C21—H21C	109.00
C15—C16—C17	108.13 (17)	H21A—C21—H21B	109.00
C15—C16—C18	110.05 (18)	H21A—C21—H21C	109.00
C12—C17—C16	112.99 (15)	H21B—C21—H21C	109.00
C1—C2—H2	119.00	C20—C22—H22A	109.00
C3—C2—H2	119.00	C20—C22—H22B	109.00
C2—C3—H3	120.00	C20—C22—H22C	109.00
C4—C3—H3	120.00	H22A—C22—H22B	109.00
C4—C5—H5	120.00	H22A—C22—H22C	110.00
C6—C5—H5	120.00	H22B—C22—H22C	109.00
			
C7—O1—C4—C3	−180.0 (2)	C1—C8—C13—C14	−74.3 (2)
C7—O1—C4—C5	0.2 (3)	C9—C8—C13—C12	−18.5 (2)
C12—O2—C10—N2	172.48 (16)	C9—C8—C13—C14	163.20 (16)
C12—O2—C10—C9	−7.6 (3)	C8—C9—C10—O2	−8.6 (3)
C10—O2—C12—C13	9.5 (3)	C8—C9—C10—N2	171.34 (19)
C10—O2—C12—C17	−171.11 (16)	C11—C9—C10—O2	176.45 (17)
C6—C1—C2—C3	−0.2 (3)	C11—C9—C10—N2	−3.6 (3)
C8—C1—C2—C3	−177.04 (18)	O2—C12—C13—C8	5.1 (3)
C2—C1—C6—C5	0.2 (3)	O2—C12—C13—C14	−176.54 (17)
C8—C1—C6—C5	177.02 (16)	C17—C12—C13—C8	−174.16 (18)
C2—C1—C8—C9	79.6 (2)	C17—C12—C13—C14	4.2 (3)
C2—C1—C8—C13	−41.5 (2)	O2—C12—C17—C16	−161.46 (16)
C6—C1—C8—C9	−97.08 (19)	C13—C12—C17—C16	17.9 (3)
C6—C1—C8—C13	141.80 (17)	C8—C13—C14—O3	0.7 (3)
C1—C2—C3—C4	0.2 (3)	C8—C13—C14—C15	−177.12 (17)
C2—C3—C4—O1	−180.0 (2)	C12—C13—C14—O3	−177.70 (19)
C2—C3—C4—C5	−0.1 (3)	C12—C13—C14—C15	4.5 (3)
O1—C4—C5—C6	179.9 (2)	O3—C14—C15—C16	147.2 (2)
C3—C4—C5—C6	0.1 (3)	C13—C14—C15—C16	−35.0 (3)
C4—C5—C6—C1	−0.1 (3)	C14—C15—C16—C17	54.3 (2)
C1—C8—C9—C10	−103.3 (2)	C14—C15—C16—C18	172.66 (18)
C1—C8—C9—C11	71.7 (2)	C14—C15—C16—C19	−66.4 (2)
C13—C8—C9—C10	20.3 (2)	C15—C16—C17—C12	−45.0 (2)
C13—C8—C9—C11	−164.75 (16)	C18—C16—C17—C12	−164.36 (18)
C1—C8—C13—C12	104.1 (2)	C19—C16—C17—C12	75.5 (2)

### Hydrogen-bond geometry (Å, º)

Cg2 is the centroid of the O2/C8–C10/C12/C13) 4*H*-pyran ring.

**Table:**

*D*—H···*A*	*D*—H	H···*A*	*D*···*A*	*D*—H···*A*
N2—H2*A*···N1^i^	0.86	2.30	3.142 (3)	168
N2—H2*B*···O3^ii^	0.86	2.19	3.029 (2)	165
C6—H6···N1^iii^	0.93	2.51	3.258 (3)	137
C18—H18*A*···*Cg*2^iv^	0.96	2.77	3.677 (2)	158

Symmetry codes: (i) −*x*+2, −*y*+1, −*z*+1; (ii) *x*+1, *y*, *z*; (iii) −*x*+1, −*y*+1, −*z*+1; (iv) *x*, *y*−1, *z*.
